# KAT3B-p300 and H3AcK18/H3AcK14 levels are prognostic markers for kidney ccRCC tumor aggressiveness and target of KAT inhibitor CPTH2

**DOI:** 10.1186/s13148-018-0473-4

**Published:** 2018-04-04

**Authors:** Elisa Cocco, Manuela Leo, Claudia Canzonetta, Serena Di Vito, Antonello Mai, Dante Rotili, Arianna Di Napoli, Andrea Vecchione, Cosimo De Nunzio, Patrizia Filetici, Antonella Stoppacciaro

**Affiliations:** 1 Surgical Pathology Units, Department of Clinical and Molecular Medicine, Ospedale Sant’Andrea, La Sapienza University, Rome, Italy; 2 Department of Biology and Biotechnology “C. Darwin”, La Sapienza University of Rome, Rome, Italy; 3 Department of Immunology, IRCCS Bambino Gesù Children’s Hospital, Rome, Italy; 4 Institute of Molecular Biology and Pathology-CNR, La Sapienza University of Rome, P.le, A. Moro 5, Rome, Italy; 5 Department of Drug Chemistry and Technology, Istituto Pasteur Italia - Fondazione Cenci Bolognetti, La Sapienza University, P.le Aldo Moro, 5, 00185 Rome, Italy; 6 Urology Unit, Department of Clinical and Molecular Medicine, Ospedale Sant’Andrea, La Sapienza University, Rome, Italy

**Keywords:** CPTH2, KAT3B-p300, ccRCC, Tumor tissues, H3-AcK18

## Abstract

**Background:**

Kidney cancer and clear cell renal carcinoma (ccRCC) are the 16th most common cause of death worldwide. ccRCC is often metastasized at diagnosis, and surgery remains the main treatment; therefore, early diagnosis and new therapeutic strategies are highly desirable. KAT inhibitor CPTH2 lowers histone H3 acetylation and induces apoptosis in colon cancer and cultured cerebellar granule neurons. In this study, we have evaluated the effects of CPTH2 on ccRCC 786-O cell line and analyzed drug targets expressed in ccRCC tumor tissues at different grade.

**Results:**

CPTH2 decreases cell viability, adhesion, and invasiveness in ccRCC cell line 786-O. It shows preferential inhibition for KAT3B-p300 with hypoacetilating effects on histone H3 at specific H3-K18. Immunohistochemical analysis of 70 ccRCC tumor tissues compared with peritumoral normal epithelium showed a statistical significant reduction of p300/H3AcK18 paralleled by an increase of H3AcK14 in G1 grade and an opposed trend during tumor progression to worst grades. In this study, we demonstrate that these marks are CPTH2 targets and significative prognosticators of low-grade ccRCC tumor.

**Conclusions:**

ccRCC is substantially insensitive to current therapies, and the efficacy of clinical treatment is dependent on the dissemination stage of the tumor. The present study shows that CPTH2 is able to induce apoptosis and decrease the invasiveness of a ccRCC cell line through the inhibition of KAT3B. In a tumor tissue analysis, we identified new prognosticator marks in grade G1 ccRCC tumors. Low KAT3B/H3AcK18 vs. high H3AcK14 were found in G1 while an opposed trend characterized tumor progression to worst grades. Our collected results suggest that CPTH2 reducing KAT3B and H3AcK18 can be considered a promising candidate for counteracting the progression of ccRCC tumors.

**Electronic supplementary material:**

The online version of this article (10.1186/s13148-018-0473-4) contains supplementary material, which is available to authorized users.

## Background

Kidney cancer is classified as the 16th most common cause of death from cancer worldwide [1]. The majority of kidney cancers (70%) are classified as clear cell renal carcinoma (ccRCC), at average age of diagnosis (60–64 years) [2]. It is often metastasized [3]; therefore, identification of new therapeutic strategies is highly desirable. ccRCC is associated with VHL loss of function with stabilization of hypoxia inducible factors (HIF-1α and HIF-2α) in both sporadic and familial forms [4]. Recent studies highlighted major roles for epigenetic regulation in the development and progression of the disease. Several epigenetic regulators such as SWI/SNF polybromo PBRM1, histone deubiquitinase BAP1, methyltransferase SETD2, and acetyltransferase MYST1 (KAT8) are significantly altered in ccRCC [5–8]. Noteworthy, global levels of histone H3 acetylation has been correlated with disease progression [9, 10] suggesting that histone modifications are tightly linked to ccRCC. The K-histone acetyl transferase KAT3B (p300) could play a role [11], and its overexpression is detected in the most aggressive cases of hepatocellular carcinoma [12]. In prostate cancer, KAT3B promotes tumor growth and activation of androgen receptor [13] weakens invasiveness in melanoma, breast, and prostate cancer cell lines [14–16]. Its nuclear localization is linked to pro-tumoral effects while cytoplasmic to a less severe outcome [17]. Epigenetic drugs are potential tools for pharmacological research and therapeutic applications. HDAC inhibitors have been extensively studied as potential anticancer treatment; however, they exhibit different effects across various renal cell lines [18]. On the other hand, there is little information on the use of K-acetyltransferase inhibitors (KATi). Nonetheless, some of them were shown to prevent growth in a broad panel of cancer cells lines [19, 20] such as neuroblastoma and glioma [21, 22]. In the past, we identified a novel KAT inhibitor, cyclopentylidene-[4-(4-chlorophenyl)thiazol-2-yl)hydrazone (CPTH2), in yeast [23]. CPTH2 was tested in different experimental models with effects on axon outgrowth [24], adenovirus infection [25], expression of superoxide dismutase in human monocytes [26], and impaired antibody response in B lymphocytes [27]. In colon adenocarcinoma, CPTH2 lowers cancer growth, decreasing GCN5 activity regulated by cMyc/E2F1 [28]. In the presented study, we assessed the effects of CPTH2 on K1 papillary thyroid and ccRCC 786-O cell lines. CPTH2 lowered KAT activity of nuclear cell extracts showing specificity for KAT3B. Importantly, it lowered cell viability, impaired invasiveness and migration, and modified cell adhesion with a global effect on cytoskeleton organization. We also analyzed the effects of the drug on the acetylation of histone H3 globally and at selected residues, and we found a hypoacetilating effects at specific H3AcK18. The results obtained by treating the cells with CPTH2 were comparable with the effects induced by silencing KAT3B, thus confirming the inhibitory selectivity of CPTH2 for KAT3B in 786-O cell line. The collected experimental results on cell lines were paralleled by a detailed analysis of KAT3B broad distribution and global levels of histone H3AcK14 and H3AcK18 in 70 ccRCC patients. Tumor and normal tissues from kidney specimens at different grades and stages were compared. Notably, we found a sudden increase of H3AcK18 and KAT3B in the switch from G1 to G2 tumor grade. Surprisingly, this effect was paired by a progressive decrease of H3AcK14 levels. This analysis suggests a novel approach and identifies promising prognosticators in clear cell carcinoma.

## Methods

### Cell cultures and treatment with CPTH2

786-O human ccRCC cell line (ATCC, Manassas, VA) was grown in RPMI 1640 plus 10% FBS, 2 mM L-glutamine, 25 U/ml penicillin, and 25 U/ml streptomycin (Gibco, Thermo Fisher Scientific, Waltham, MA). K1 human thyroid carcinoma cell line (ECACC, Sigma-Aldrich, St. Louis, MO) was cultured in DMEM:Hamm’s F12:MCDB 105 medium (2:1:1) plus 10% FBS, 2 mM L-glutamine, 25 U/ml penicillin, and 25 U/ml streptomycin (Gibco, Thermo Fisher Scientific). Cells were maintained in a humidified atmosphere of 5% CO_2_ at 37 °C. Cyclopentylidene-[4-(4chlorophenyl) thiazol-2-yl)hydrazone (CPTH2) was dissolved in 100 mM/L dimethyl sulfoxide (DMSO; Sigma-Aldrich) and diluted to the final concentrations in complete medium. For all the experiments, cells were treated with 1% DMSO as control. After 24 h from seeding, exponentially growing 786-O and K1 tumor cells were treated with CPTH2 at concentrations ranging from 1 to 200 μmol/L for 24 to 120 h; 24–48 h culture and 100 mM/L were then chosen as CPTH2 concentration giving the strongest growing inhibitory effects without signs of cell damage or exhaustion of the drug function [29].

### CPTH2 stability

2.9 mg of CPTH2 (MW 291.80) have been dissolved in 0.3 ml of DMSO and then diluted with PBS buffer (pH = 7.4) to 2 ml (5 mM). Then, the resulting solution was incubated at 37 °C on a heating block and checked for purity after different times (30 min, 1, 2, 12, 24, 36, 48, 72, and 132 h) by Thin Layer Chromatography (TLC) on silica gel plates eluting with the mixture Ethyl Acetate:Petroleum Ether (1:2). Over time, no traces of degradation products were detected by TLC. CPTH2 is stable in PBS buffer at 37 °C since no traces of degradation products can be detected by TLC up to 132 h of incubation***.***

### In vitro HAT assay

Histone acetyltransferase activity was measured with fluorescent in vitro HAT Assay Kit (Active Motif, CA) in nuclear extracts (7 μg) prepared with EpiQuik™ Nuclear Extraction Kit I (Epigentek, NY) treated 24 or 48 h with CPTH2 100 μM or solvent DMSO. Twenty-five micrograms of recombinant proteins p300 (Active Motif), GCN5 (Active Motif), and PCAF (Biomol, DE) were incubated with Anacardic Acid 15 μM, CPTH2 400 μM, and 600 μM or in DMSO. HAT activity was measured in tubes in presence of Ac-CoA (0.5 mM) and histone H3 (50 μM). Resulting fluorescence was measured with GloMax® (Promega, WI) after conjugation between developer and free sulfhydryl groups on CoA-SH.

### Cell viability and apoptosis assay

Percentage of viable cells was evaluated by trypan blue dye exclusion method. After monolayer cell trypsinization (0.25 mg/ml trypsin, Gibco, Thermo Fisher Scientific), cells were stained with trypan blue (0.04%, Gibco) for 2 min, and vital, unstained cells were counted with emacytometer. The percentage of unstained cells was obtained as mean ± SD of four independent experiments. 786-O cell apoptosis was assayed with MuseAnnexinV and Dead Cell kit (Millipore, Darmstadt, DE); 1 × 10^5^ cells from untreated, DMSO and CPTH2 samples were centrifuged (2000 rpm, 5 min), washed in PBS, and resuspended in PBS plus 1% BSA (Sigma-Aldrich) 1% FBS, with 100 μL of Dead Cell reagent containing AnnexinV and 7-aminoactinomycin D (7-AAD). After 20 min RT incubation at room temperature in the dark, cells were applied to a Muse Cell Analyzer (Millipore), and the results are expressed as percentage of apoptotic cells ± SD.

### In vitro migration, invasiveness, and adhesion

Migratory and invasive capacities of 786-O cells were evaluated using the BioCoat Invasion Chamber system (BD Biosciences, San Jose, CA). Matrigel invasion chambers, containing an 8-μm-pore-size PET membrane, were treated with Matrigel Basement Membrane Matrix (invasion test; BD Bioscience) or with BSA (migration test; Sigma); ~ 1.5 × 10^5^ cells diluted in 0.2% FBS-DMEM were added to upper compartment and 2.5 ml 10% FBS-DMEM to the lower compartment. Migration assay was performed for 24 and 48 h in a humidified tissue culture incubator at 37 °C in a 5% CO_2_ atmosphere. After incubation, no migrating cells were removed by scrubbing the upper face of the membrane, and migrated cells present on the lower surface of the membrane were stained with Diff-Quick; cells present in 10,400× enlargement field were counted in each filter. The data are given as mean ± SD of triplicate filters. For adhesion test, a 96-well plate was coated with BSA (5 mg/ml, Sigma-Aldrich), fibronectin (5 μg/ml, bovine, Calbiochem), matrigel (3 mg/ml, standard matrigel matrix, BD Biosciences), and collagen type IV (5 μmg/ml, bovine, Sigma-Aldrich). Cells treated for 24 and 48 h with CPTH2 or DMSO were plated 5 × 10^4^ on each coated well in triplicate and incubated to allow adhesion at 37 °C for 1 h. After PBS washing, cells were fixed in ice-cold acetone/ethanol (1:1, Sigma-Aldrich) for 10 min and washed with 20% methanol. Cells stained with crystal violet solution (0.5% *w*/*v* in 20% methanol, Sigma-Aldrich) were measured in a spectrophotometer at 540 nm (Multiskan spectrum, Thermo) after color solubilization with 0.1 M sodium citrate pH 4.2 (50% EtOH, Sigma-Aldrich).

### Scratch assay

Cell migration was tested with “wound healing” assay [30]. Briefly, 786-O cells were seeded in a 6-well plate and cultured until confluence, scraped with a 200-μl micropipette tip, then incubated with CPTH2 (100 μM), DMSO, or RPMI; the growth was photographed at 0 and 48 h with an inverted microscope (Nikon Eclipse TE2000-S) and digital camera (Nikon Coolpix S4, 6.0 Mpix, 10× zoom). Wound area was measured and quantified with TScratch Software [31].

### RNA interference

18-20 h before transfection, 786-O were plated in 6-well plates in complete growth medium; at 60% of confluency, cells were placed in OptiMEM (serum-and antibiotics-free medium; Thermo Fisher Scientific) and transfected with 30 nM of p300 small interfering RNA (HSC.RNAII.N001429.12.1, IDT, San Jose, CA) or Negative Control 1 (IDT) using Lipofectamine 2000 according to the manufacturer (Invitrogen, Thermo Fisher). Six hours after transfection, the medium was changed to full growth conditions, and cells were harvested at 6, 12, 24, and 48 h post-transfection. p300*si* efficiency was assessed by real-time PCR transcript analysis of p300 mRNA.

### Immunofluorescence

786-O cells were seeded on glass coverslips in 35 mm Petri dishes and cultured until 50% confluence. They were treated with CPTH2 (100 μM) for 18 h or transfected with 30 nM si-p300 for 24 h, washed three times with PBS and fixed with 4% paraformaldehyde (PFA; Sigma-Aldrich) in PBS, permeabilized with 0.2% Triton X-100 (Sigma-Aldrich), and blocked with 1% BSA. Then, they were incubated with rhodamine–phalloidin (1:1000, Thermo Fisher Scientific) in 2% BSA in PBS for 1 h, washed with PBS, and stained with DAPI (1:10000, 1 mg/mL stock solution, Roche, Basel, CH). Images were acquired with the Nikon fluorescent microscopy, and stress fibers were counted by analyzing 100 cells in different fields for each experimental point. The data are given as mean ± SEM of stress fiber numbers per cell.

### RNA isolation and real-time PCR analysis

Total RNA was isolated from 786-O cell line with TRIzol reagent (Ambion, Thermo Fisher), quantified with Nanodrop 2000 (Thermo Fisher). Two hundred fifty nanograms of total RNA were reverse transcribed with High-Capacity RNAtocDNA Reverse Trascription kit (Applied Biosystems, Thermo Fisher). Real-time PCR was performed in Stratagene Mx3005P (Agilent Technologies, Santa Clara, CA) with TaqMan2X Universal Master Mix (Applied Biosystems) in 20 μl mixture. Each sample assayed in triplicate was performed with PCR cycles: (10 min) at 95 °C and 60 cycles of (15 s) at 95 °C and a final (1 min) at 60 °C. The primers and probes of the following transcripts were EP300 (Hs00914223_m1), AKT-1 (Hs00178289_m1), TGF-b2 (Hs00234244_m1), HIF-1a (Hs00153153_m1), CD44 (Hs01075864_m1), ITGb1 (Hs01127536_m1), ITGb3 (Hs01001469_m1), ITGa5 (Hs01547673_m1), and ITGa6 (Hs01041011_m1) (Applied Biosystems). The fold change of gene expression was calculated using the 2-ΔΔCT method, and all values were normalized to endogenous control ACTB (Hs 99999903_m1, Applied Biosystems) and expressed in arbitrary units.

### Bulk histone preparations and western blot analysis

Cells were seeded at 200,000 per well, after 24 h were treated with CPTH2, DMSO, p300*si*, NC1 and incubated at 37 °C for 12, 24, and 48 h in a humidified atmosphere of 5% CO_2_. Total protein extracts were resuspended in Laemmli buffer (Bio-Rad, CA) and heated 5 min at 90 °C. Protein extracts were run on 15% SDS-PAGE, blotted onto nitrocellulose (GE Healthcare Life Sciences, UK) and hybridized with anti-H3AcK18 (Abcam, UK), anti-H3Ac (Merck, Germany), and anti-GAPDH (Santa-Cruz, TX) antibodies. Fluorescence detected by Long Lasting Chemilumiscent Substrate (EuroClone, Italy) was visualized by ChemiDoc™ MP Imaging System (Bio-Rad).

### Tissue samples and immunohistochemistry

Clear cell RCC tissues and matched normal adjacent tissues were collected from 70 patients (listed in Table 1) with primary ccRCC between January 2008 and December 2014, who underwent kidney tumor radical surgery at the Sant’Andrea Hospital of Roma “La Sapienza” University. The use of the histological material was authorized by personal patient consensus according to S. Andrea Hospital policy form. Patient medical records including tumor staging, pathological diagnosis, and surgical data were reviewed and classified according to the American Joint Committee on Cancer [32]. Formalin-fixed and paraffin-embedded ccRCC tissue blocks were sectioned, deparaffinized in xylene, and rehydrated through a graded ethanol series and then subjected to antigen retrieval by boiling in 0.01 M sodium citrate buffer (pH 6) 10 min in microwave. Endogenous peroxidase was blocked for 10 min in 3% hydrogen peroxide in methanol, incubated 1 h RT with primary antibody diluted to anti-p300 rabbit polyclonal antibody 1:1000 (Bethyl Laboratories), anti-H3AcK18 1:2000 (Abcam), and anti-H3AcK14 1:2000 (Abcam). Reactions were followed with DAB detection kit (Dako). Immunostaining results were recorded as percentage of positive cells in increments of 10% regardless of the intensity of the staining. Cases were considered as negative if < 5% of tumor cells were positive. Immunohistochemical and morphological analyses were evaluated by pathologists (AS and AV).

**Table 1:** Patients characteristic

	Number of cases	Age ± SD (range)	Tumor grade [41] (WHO-UISP2011)	Tumor stage [32] pTNM
Cases	70	64.21 ± 11.24 (29–91)	G1 = 20 G2 = 26 G3 = 24	Stage 1 = 35Stage 2 = 16Stage 3 = 16Stage 4 = 1
Male	46	64.63 ± 11,84 (29–91)	G1 = 15 G2 = 16 G3 = 15	Stage I = 23Stage II = 9Stage III =12Stage IV = 1
Female	24	63.42 ± 10.18 (45–79)	G1 = 5 G2 = 10 G3 = 9	Stage I = 12Stage II = 7Stage III = 4Stage IV = 0

### ICC

10^4^ si-p300 cells untreated and treated in DMSO w/w CPTH2 were spotted on glass slide using cytocentrifuge (Cytospin3 Seongkohn traders, Korea) at 700 rpm for 5 min; slides were fixed with cold acetone for 15 min, allowed to dry, and stored in PBS at 4 °C. The cell spots were incubated with primary antibody as previously described.

### Statistical analysis

Experiments were performed in triplicate and results recorded. Cell line data were presented as mean ± standard deviation (SD). Statistical analysis was performed using the S-PSS 12.0 software. Evaluation of data distribution showed a non-normal distribution of the study data set. Differences between groups of patients in medians for quantitative variables and differences in distributions for categorical variables were tested with the Kruskal–Wallis one-way analysis of variance and chi-square test, respectively. Using multiple logistic regression with the enter method, variables as assessed in the univariate analysis were entered and investigated as predictors of ccRCC G2-G3 versus G1 and in a separate model predictors of high stage (stages II–III) versus low stage (stage I) were compared. The logistic regression analysis was carried out using the data from patients for whom complete data were available. The variables considered for entry into the model included age, p300, H3AcK14, and H3AcK18. An alpha value of 5% was considered as the threshold for significance. The data are presented as mean ± standard deviation (SD); in vitro HAT assay, RT-PCR, and western blot analysis were presented as mean ± SEM. Student *t* test was calculated, and *p* value ≤ 0.05 was considered significant. Odds ratios and 95% CIs were calculated for the parameters in each group using ccRCC G1 and ccRCC stage I as a reference group.

## Results

### CPTH2 inhibits HAT activity and decreases tumor cell viability through apoptosis

**Fig. 1:**
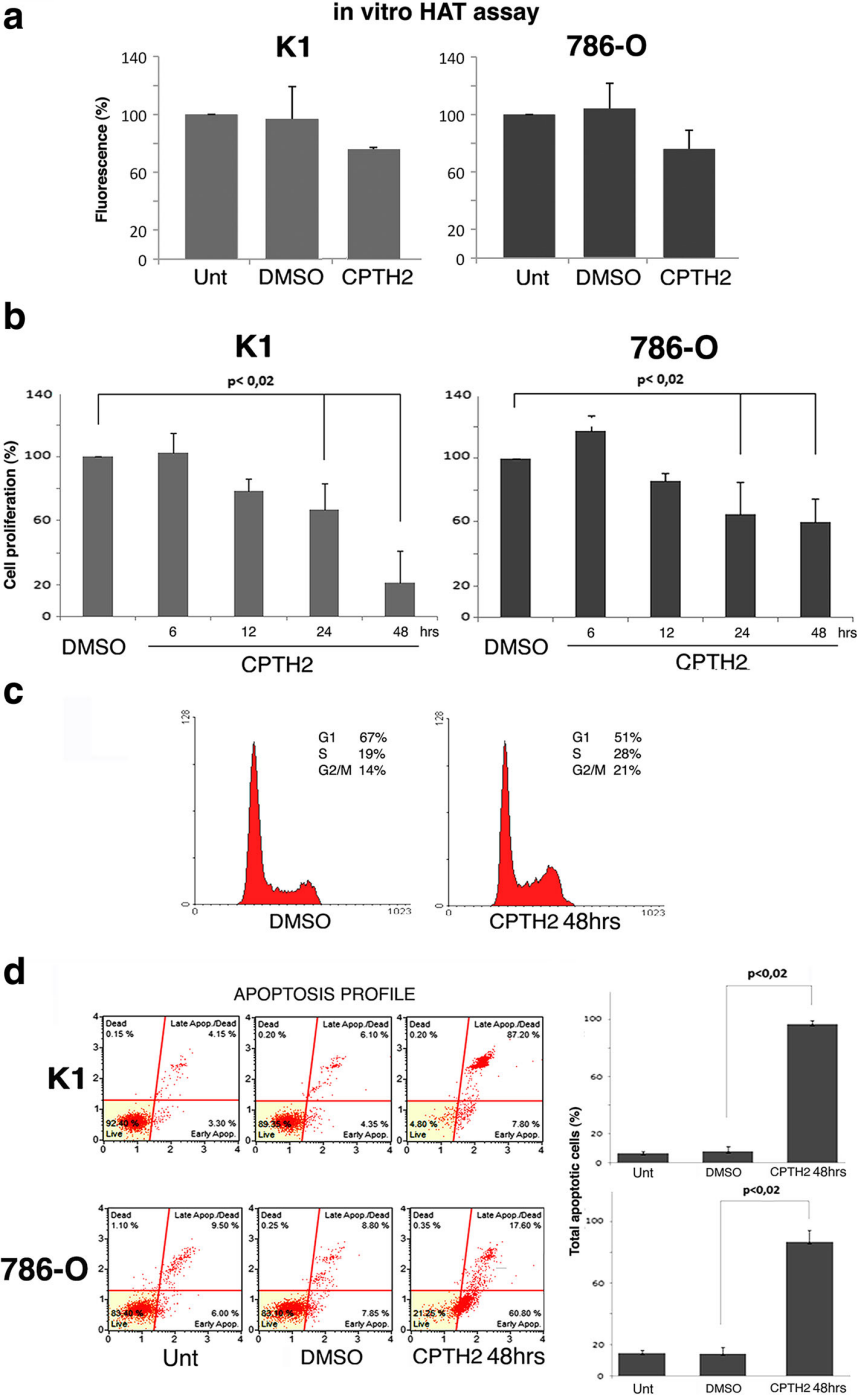
HAT inhibitor CPTH2 decreases cell viability in tumor cells. **a** In vitro HAT activity was measured in nuclear extracts of Thyroid papillary K1 (gray) and clear cell renal carcinoma ccRCC 786-O (dark gray) cell lines treated with CPTH2 (100 μM) for 24 and 48 h, respectively, and compared to untreated and DMSO samples. **b** Cell proliferation measured in K1 (gray) and 786-O (dark gray) cell lines in DMSO or during CPTH2 treatment at increased times (h) showed strong reduction at 48 h. **c** FACS analysis of 786-O cell line grown in DMSO and treated for 48 h with CPTH2. **d** Apoptotic FACS profile and percentage of apoptotic cells strongly enhanced after 48 h CPTH2 treatment in both K1 and 786-O cell lines

Papillary thyroid (K1) and clear cell Renal Cell Carcinoma (ccRCC-786-O) cell lines were incubated with CPTH2 at the most effective concentration of 100 μM in comparison with untreated and excipient (DMSO) controls (Fig. 1). In vitro HAT activity was measured in 24 h K1 and 48 h 786-O treated cells, respectively, depending on the responsiveness of individual cell lines. Despite intrinsic levels of HAT activity been higher in K1 than in 786-O, CPTH2 treatment caused a comparable drop of the activity in both lines (Fig. 1a), according to a direct enzyme’s inhibition of the drug. To investigate the capacity of CPTH2 to affect cell proliferation, cells were treated in DMSO *w*/*w* CPTH2 for 12, 24, and 48 h and washed and the number of cells adjusted by day exclusion and cultured in medium. CPTH2 treatment caused a decrease in cell proliferation after as early as 12 h with a further significant reduction after 48 h stimulation. K1 cell line, which is derived from papillary thyroid carcinoma and is responsive to chemotherapy and apoptotic drugs, showed a reduction of 80% after 48 h. ccRCC-786-O, which is from renal clear cell carcinoma and is much less sensitive to anti-proliferative drugs, presented a significant, but less pronounced, decrease, 40% (Fig. 1b). In order to demonstrate whether CPTH2 affected the cell cycle FACS analysis was performed confirming that no relevant defects in cell cycle progression were induced in ccRCC 786-O cells after 48 h treatment with CPTH2 (Fig. 1c). CPTH2 was also unable to change cell cycle progression in ccRCC 786-O and K1 papillary thyroid cells treated with prolonged treatment (Additional file 1). Accordingly, no substantial differences were obtained when 786-O cells were treated for 24 and 48 h in DMSO *w*/*w* CPTH2 as shown in the immunostaining with Ki67 and Cyclin D1 antibodies (Additional file 2). Annexin-V FACS analysis of K1 and 786-O cell lines showed in addition that CPTH2 treatment produced a drastic increase in apoptotic/dead cell population after 48 h quantified as a percentage of total apoptotic cells (Fig. 1d and Additional file 1), suggesting that CPTH2 treatment leads to cell death rather than cell cycle arrest. Collectively, these results indicate that the KAT inhibitor CPTH2 is active on both thyroid papillary K1 and 786-O cell lines, inhibits in vitro HAT activity in nuclear extracts, and lowers cell viability after 48 h treatments, thus leading to cell death.

### CPTH2 changes the cytoskeleton organization of ccRCC-786-O cells and decreases invasiveness and migration

**Fig. 2:**
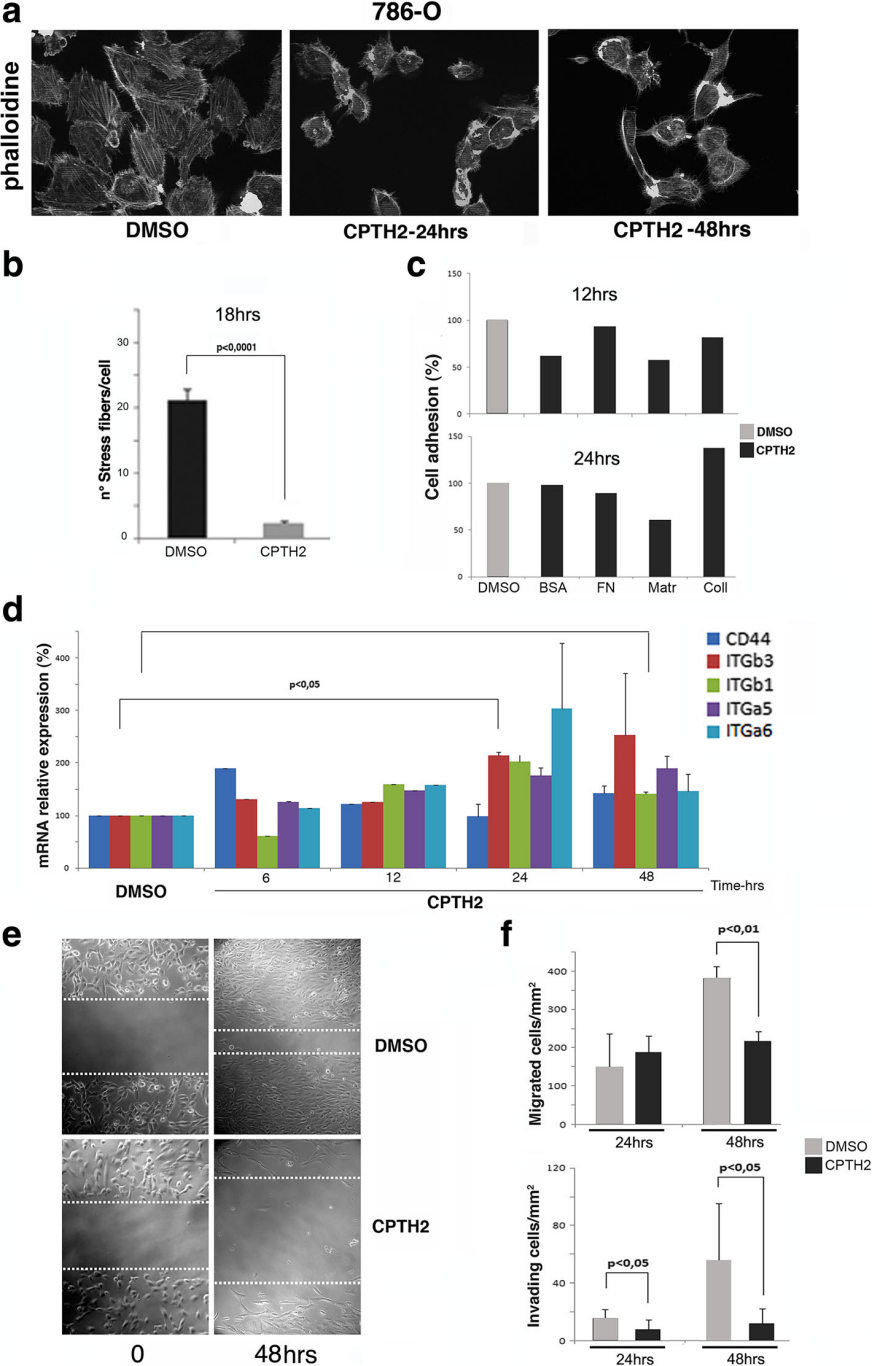
CPTH2 modifies stress fiber organization and cell morphology of ccRCC 786-O cell line. **a** Cytoskeleton organization stained with phalloidine showed a drastic reorganization of stress fibers, adhesion placks, and cell morphology after 48 h of CPTH2 (100 μM) treatment with respect to untreated and DMSO samples. **b** Percentage of stress fibers/cell after 18 h of CPTH2 treatment compared to DMSO sample. **c** Percentage of 786-O cell adhesion (%) after 12 and 24 h CPTH2 treatment with respect to DMSO tested on different substrates, BSA, fibronectin (FN), matrigel (Matr.), and collagen (Coll). **d** mRNA expression of modulators of adhesion and migration CD44, and integrins ITGb3, ITGb1, ITGa5, and ITGa6 analyzed by qRT-PCR, shows remarkable similarity with an increase after 24/48 h of CPTH2 treatment. **e** Scratch test confirmed a strong inhibition of cell migration and invasiveness after 48 h of CPTH2 treatment compared to DMSO control. **f** Percentage of migrating (cells/mm^2^) and invading cells (cells/mm^2^) after 24 and 48 h of CPTH2 (black) treatment with respect to DMSO (light gray)

The capability of a novel compound to counteract invasion and metastatic growth is a fundamental feature to be considered in the development of new potential antitumor drugs. We therefore analyzed the effects of CPTH2 against the invasive properties of 786-O cell line. We treated cells at increasing time with CPTH2 (100 μM); after 24 and 48 h, we observed an evident reduction of cell volume and a gross rearrangement of cytoskeleton organization with conglobation and disaggregation of the actin stress fibers and retraction of organized phila as evidenced by the phalloidin-TRITC staining (Fig. 2a). The number of visible actin stress fibers was already strongly lowered after 18 h of drug treatment suggesting a modulation of cell adhesion (Fig. 2b). We next assayed the adhesion capabilities of 786-O cells grown on different substrates treated with CPTH2 for 12 and 24 h. Interestingly, CPTH2 was able to modulate cell adhesion only when 786-O cells were plated on the complex synthetic matrix Matrigel, while on single components such as fibronectin and collagen, it was substantially unaffected (Fig. 2c). This effect was evident after 48 h treatment hinting that the reduced adhesiveness of 786-O tumor cells is likely dependent on the concerted modulation of several different adhesion molecules. We next asked whether genes known to be involved in adhesion and migration such as integrins were affected at transcriptional level. mRNA expression profiles of selected marker genes such as the transmembrane glycoprotein CD44, involved in lymphocyte migration and metastasis [33] and integrins ITGb3, ITGb1, ITGa5, and ITGa6 [34] that were comparatively analyzed by RT-qPCR (Fig. 2d). The summary panel shows an overall coordinated upregulation after 24 and 48 h of treatment, indicating a clear deregulation of adhesion genes and a coordinated behavior induced by drug treatment. This result is in agreement with effects in the deregulation of integrins. It is known, in fact, that their up or downregulation [35, 36] is responsible to modulate cell invasion and migration in cancer microenvironment. We finally confirmed the inhibitory activity of CPTH2 on the cell invasiveness properties, performing an in vitro wound healing scratch tests. 786-O cells were scraped and allowed to grow and migrate in CPTH2 versus DMSO (Fig. 2e). Results indicate that after 48 h, it is clearly seen that while cells in DMSO were actively migrating and able to fill the gap in the presence of CPTH2 migration was severely inhibited. Migration and Matrigel invasion were tested in Boyden chambers. Figure 2f shows results, migration (upper panel), and invasion (lower panel) were evaluated after 24 and 48 h in DMSO *w*/*w* CPTH2 showing a drastic decrease after 48 h of treatment. Collectively, our data demonstrate that CPTH2 is capable to counteract invasion and migration of 786-O cells in culture.

### CPTH2 shows selectivity for KAT3B-p300

**Fig. 3:**
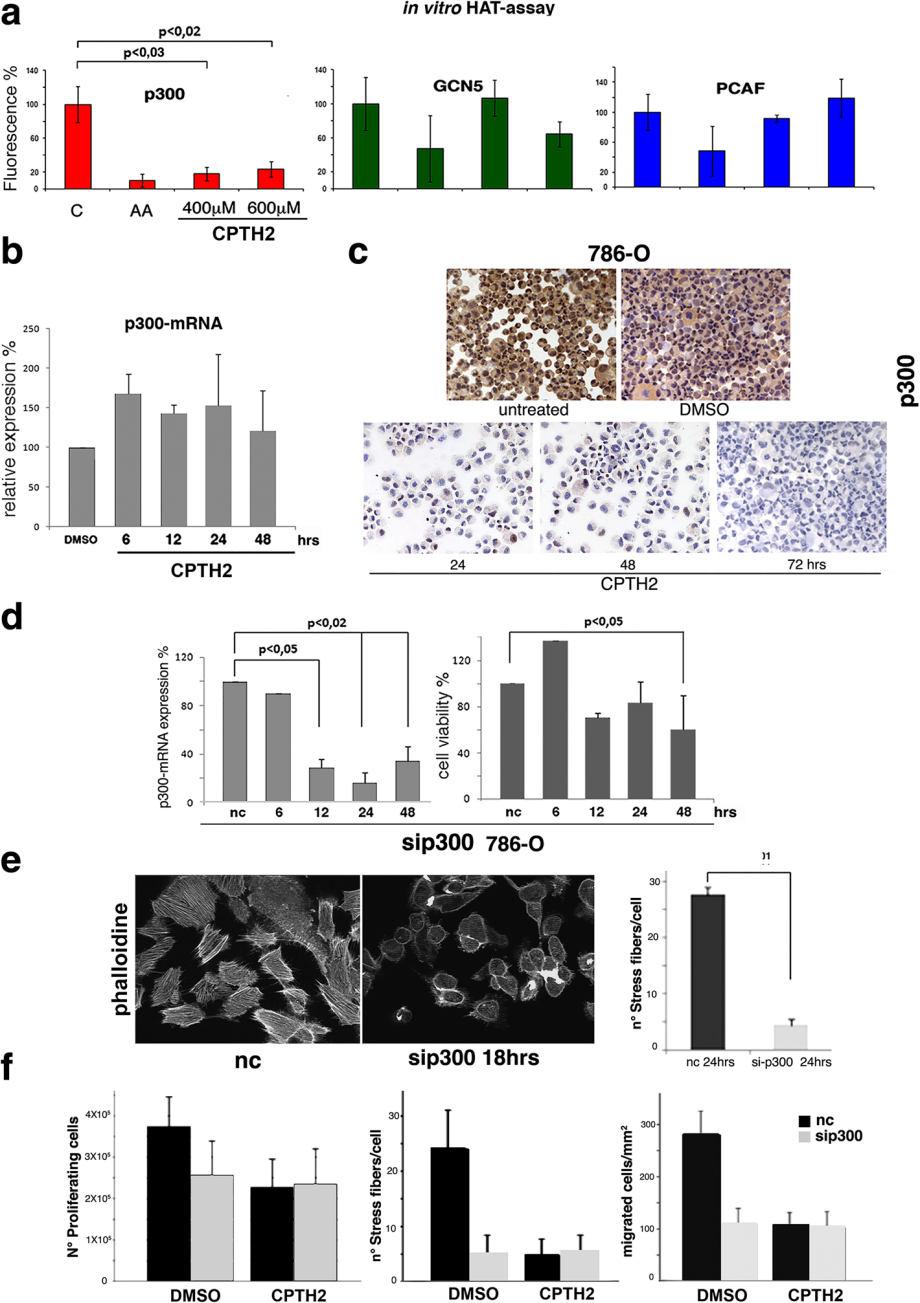
CPTH2 preferentially inhibits HAT-p300 in ccRCC 786-O cells. **a** In vitro HAT assay was performed on recombinant p300 (red), GCN5 (green), and PCAF (blue). Control (**c**) in presence of HAT inhibitor anacardic acid, AA (15 μM), and CPTH2 (400 and 600 μM). CPTH2 shows higher selectivity in the inhibition of p300. **b** Prolonged incubation with CPTH2 does not affect mRNA expression of p300. **c** treatment of ccRCC 786-O with CPTH2 (100 μM) at indicated times clears the immunostaining of p300 with respect to untreated and DMSO cells. **d** Silencing of p300 in 786-O cells caused decrease of p300-mRNA expression (light gray) and lowering of cell viability (gray) with respect to nc control. **e** Cytoskeleton of 786-O cells silenced for p300 (18 h) was stained with phalloidine. It is heavily modified and fully comparable to the cytoskeleton of cells treated with CPTH2 shown in Fig. 2a, and histogram with percentage of stress fibers/cell found after 24 h p300*si* (light gray) compared to control, nc (dark gray). **f** Histograms reporting the number of proliferating cells, percentage of stress fiber/cell and number of migrating cells in, respectively, nc control (black) and p300*si* cells (gray) grown in DMSO and in CPTH2 for 48 h

In order to evaluate the selectivity of CPTH2 in the inhibition of individual KATs, we carried out an in vitro HAT assay with recombinant p300, GCN5, and PCAF in the presence of increasing concentrations of CPTH2 (400 and 600 μΜ) and the known KAT inhibitor anacardic acid [20, 37]. CPTH2 preferentially inhibited p300 compared to GCN5 while it did not affect PCAF (Fig. 3a). Starting from this evidence, we first analyzed the effects of CPTH2 on p300 mRNA expression in 786-O cell line, and RT-qPCR in 6 to 48 h time lap cultures showed that p300 mRNA expression was unaffected by CPTH2 treatment at transcriptional level (Fig. 3b). We next asked whether CPTH2 may act at the protein level. Since p300 can be expressed both in the cell nucleus and cytoplasm, immunocytochemistry was performed using anti-p300 antibodies on 786-O (Fig. 3c) and K1 thyroid cultures (Additional file 3) in untreated, DMSO control, and after 72 h of CPTH2 treatment, to better depict the eventual subcellular change of expression. Surprisingly, p300 staining decreased progressively to reach an almost complete clearing after 72 h of CPTH2 stimulation in both cell lines. Our analysis also revealed that CPTH2 lowered p300 protein levels on both its nuclear and cytoplasmatic localization. We wanted to assay the effects of CPTH2 on p300 in presence of proteasomal degradation. Cells were pretreated with MG 132 for 1 h and then grown in DMSO *w*/*w* CPTH2; no relevant differences in the number of apoptotic cells nor in immunostaining intensity of p300 were obtained. These results suggest that the proteasome has not a relevant function in the decrease of p300 upon CPTH2 treatment. These results suggested that, even on live cells, CPTH2 may exert its main inhibitory activity on KAT3B-p300 and is able to lower the protein level independently from its cellular localization. On the basis of CPTH2 selectivity for KAT3B, we decided to investigate the effects of p300 silencing in 786-O cells. Figure 3d shows that KAT3B-p300 mRNA interference (left panel) was followed by an early drop of 786-O cell viability (right panel) comparable in value to the effect induced by treatment with CPTH2 100 μM (Fig. 1b vs. Fig. 3d). Furthermore, p300 silencing after 18 h resulted in a remarkable cytoskeleton rearrangement with clear drop in the number of stress fibers per cell (Fig. 3e). To provide a further demonstration that CPTH2 and p300*si* act through the same target, 786-O cells transfected with control (nc) and si-p300 RNA were grown in DMSO and treated with CPTH2 for 48 h (Fig. 3f). We found that cell proliferation, number of stress fibers, and migration showed the same values in controls and si-p300 cells, thus demonstrating full overlap between CPTH2 activity and silencing of p300. Collectively, all these results demonstrate the selectivity of CPTH2 in the inhibition of KAT3B-p300.

### CPTH2 treatment and p300*si* show similar effects on histone H3K18 acetylation and expression of selected cancer markers

**Fig. 4:**
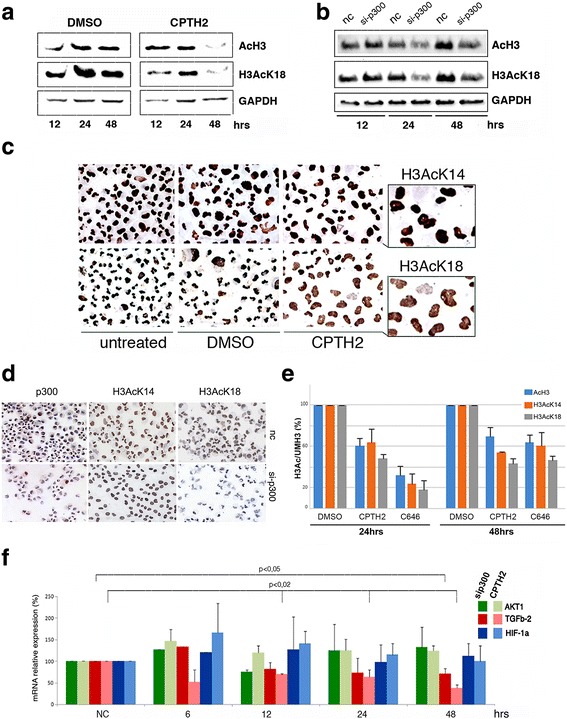
Treatment with CPTH2 and p300*si* in ccRCC 786-O cells show similar effects. **a** Western blot analysis of bulk histone preparations extracted from 786-O cells at increasing times of CPTH2 (100 μM) treatment sequentially hybridized with anti-AcH3, anti-H3AcK18, and anti-GAPDH as internal standard. **b** The same experiment performed in 786-O treated for p300*si* at increasing times in comparison with untreated nc. **c** Immunohistochemical staining of selective histone H3AcK14 and H3AcK18 in 786-O cells untreated, in DMSO and treated with CPTH2 (100 μM) for 48 h. **d** The same analysis performed in 786-O cells after 48 h p300*si* and nc control, hybridized with anti-p300, anti H3AcK14, and anti H3AcK18. **e** Histograms show the level of AcH3, H3AcK14, and H3AcK18 normalized to unmodified UMH3 found in 786-O cells treated with 24 and 48 h with CPTH2 (100 μM) and p300 inhibitor C646 (10 μM). **f** Comparison of mRNA levels of tumor markers AKT1 (green), TGFb-2 (red), and HIF-1α (blue) carried out by RT-qPCR in cells treated at increased indicated times for p300*si* (heavy colors) and with CPTH2, (100 μM), (light colors)

Increased expression of KAT3B correlates with higher acetylation of histone H3K18 in the axonal regeneration of optic nerve [38] while its and CREBB knockdown led to a global decrease of H3AcK18 in human embryonic stem cells [39]. In budding yeast, we showed a selective acetylating activity of KAT2A on H3K18 [40]. Following this line of results, we next asked whether CPTH2 might similarly affect acetylation of histone H3 and at selected H3K18 in 786-O cells. Western blot analysis of bulk histone preparations from 786-O cells were serially hybridized with anti-AcH3 and anti-H3AcK18 antibodies after a 48-h time course incubation with CPTH2 showing a reduced acetylation of both global AcH3 histone and H3AcK18 (Fig. 4a). Next, based on the matching between CPTH2 treatment and p300 silencing, we tested the global levels of AcH3 and H3AcK18 at different time points during p300*si* treatment in 786-O confirming the decrease of AcH3 and H3AcK18 after 24 h (Fig. 4b). Immunohistochemical staining of 786-O for specific H3 lysines, H3AcK14, and H3AcK18 was then performed in cells treated for 48 h with CPTH2 (Fig. 4c). Strikingly, while the staining pattern of H3AcK14 was unaltered by CPTH2 treatment, the staining of H3AcK18 was drastically weakened in comparison to control and DMSO cells, suggesting that the inhibitory activity of CPTH2 was highly selective for histone H3K18 residue with respect to H3K14. The immunohistochemical analysis on p300*si* 786-O cells with anti-p300, as a control, anti-H3AcK14, and H3AcK18 compared to untreated nc cells (Fig. 4d) showed substantially the same result. This unbiased result confirms that silencing of p300 and CPTH2 treatment induce similar effects lowering the degree of global histone H3AcK18 in a p300 dependent way. We then added a control experiment by comparing the activity of the previously described specific KAT3B inhibitor, C646 [14] with CPTH2. Western blot analysis showed that the effects of C646 and CPTH2 on inhibition of bulk AcH3, H3AcK14, and H3AcK18 were fully comparable at increasing times (Fig. 4e). To reinforce this point, we finally compared the transcriptional effects of p300*si* and CPTH2 treatment on the mRNA expression of three oncogenes: AKT1, TGFb2, and HIF-1a at different time points (Fig. 4f). The summary diagram of collected results showed convincingly that the expression trend of each gene in time is highly comparable in the two experimental conditions providing an additional conclusive demonstration on the convergent effects obtained by treatment of CPTH2 and silencing p300.

### Expression of p300, H3AcK18, and H3AcK14 of ccRCC ex vivo carcinoma

**Fig. 5:**
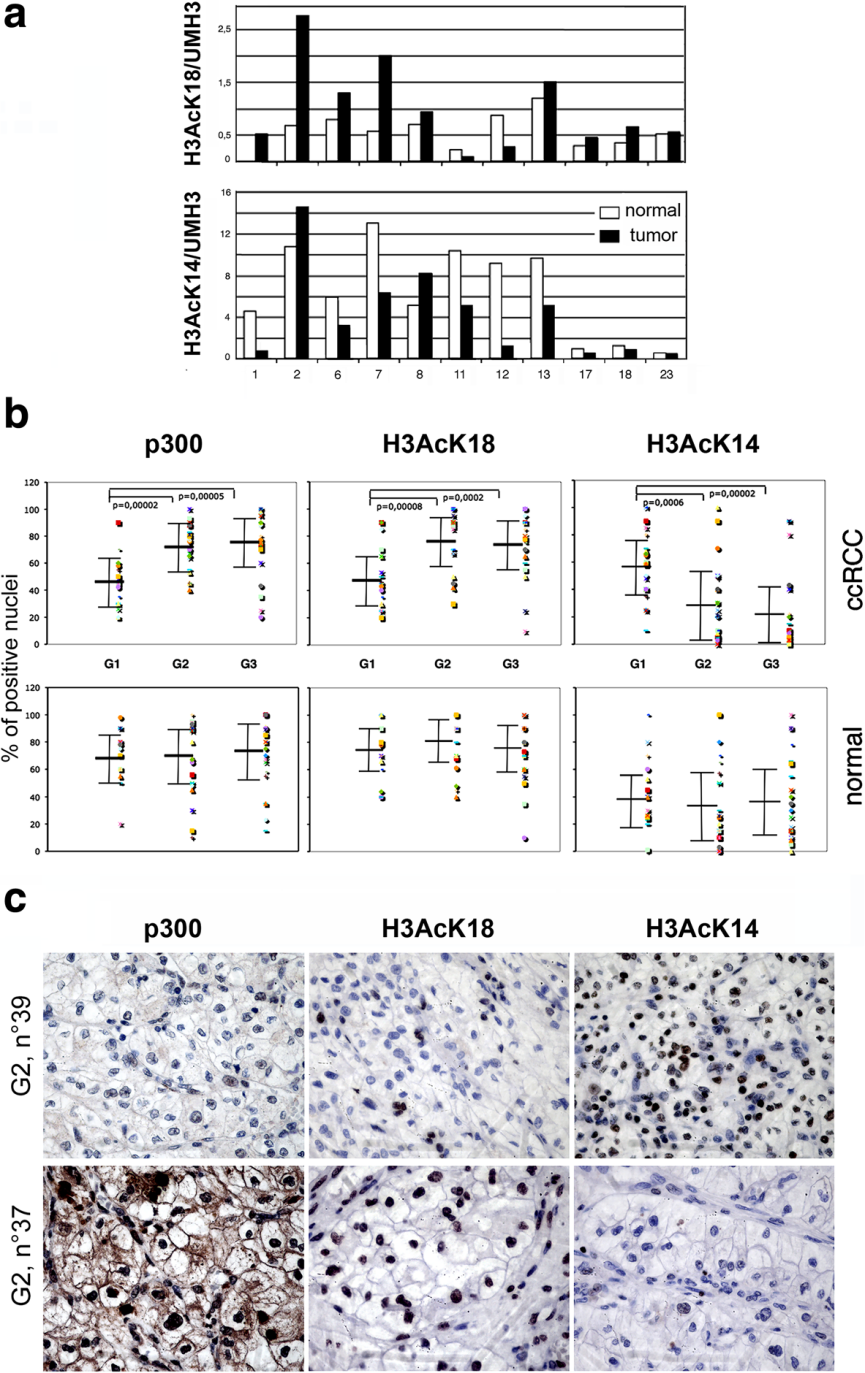
HAT p300, histone residues H3AcK14, and H3AcK18 mark renal clear cell carcinoma tumor grade progression. **a** Histograms show the level of histone H3AcK18 and H3AcK14 normalized to unmodified UMH3 found in normal (white) and tumor tissues (black) in ccRCC patients at different grade. **b** Histograms show the percentage of positive nuclei, evaluated in parallel by immunohistochemistry with anti-p300, anti-H3AcK18, and anti-H3AcK14 antibodies in cancer and paired normal tissue sections from 70 ccRCC specimen (Table 2). When grouped by differentiation grade (G1, G2, and G3), p300 and anti-H3AcK18 show a symmetrical and significant lower percentage of positive nuclei at G1 with respect to G2-G3 ccRCC and to their paired normal tissues. On the contrary, H3AcK14 displays significant increase of positive ccRCC cells at G1 decreasing through G2 and G3 versus the percentage found in the paired normal tissue. **c** Immunostaining of two representative G2 ccRCC cases (nos. 39 and 37) showing opposing behavior of p300 and H3AcK18 vs H3AcK14 expression. High nuclear expression of p300 is accompanied by a cytoplasm positivity in case no. 37. Avidin Biotin Immuno-peroxidase staining; enlargement 400×

To analyze whether ccRCC cells selected acetylated forms of histone H3K18 and H3K14, bulk preparations of histone nuclear proteins were extracted from 11 ccRCC specimens and from normal adjacent tissues and tested by western blot. The results were normalized to the amount of unmodified H3 and expressed as arbitrary units (Fig. 5a). As shown, the amount of H3AcK18 was generally much lower than H3AcK14, and overall, the acetylation of both was extremely variable among cases. Interestingly, however, the respective acetylation of tumor versus normal paired tissue was higher for H3AcK18 and lower for H3AcK14. Furthermore, G3-stage II (nos. 6 and 7), G2-stage I (nos. 13 and 23), and G3-stage I (nos. 17 and 18) [32, 41] were characterized by higher content of H3AcK18 and lower content of H3AcK14 in tumor with respect to normal paired tissues. Taken together, these data evidenced a global heterogeneity in the levels of H3AcK14 and H3AcK18 among individuals but hinted of an opposite influence of H3AcK18 and H3AcK14 residues on tumor growth. To test the hypothesis of a reciprocal behavior between H3AcK14 and H3AcK18 and the possible correlation between p300 and H3AcK18 expression as found in vitro on 786-O cell line, we decided to extend the analysis to tumor specimen from ccRCC patients after a careful tumors reevaluation. Overall, 70 ccRCC patients were entered in the study: 46 (65%) males and 24 females (35%). The mean age was 64.2 ± 11.2 years. Twenty patients (29%) present a grade 1 and 50 grades 2–3 (71%); 35 patients a stage I (50%) and 35 at stages II–III (50%) [32, 41] (Table 1). Sections from 2 to 4 different blocks for each case of ccRCC, everyone comprising normal peritumoral renal parenchyma, were immunostained with H3AcK18, H3AcK14, and KAT3B-p300 antibodies, and the results were reported as the mean percentage of stained nuclei. The choice of antibody experimental settings and parameters were chosen in order to exclude any cytoplasm staining, thus avoiding misleading results. More importantly, to avoid differential conditions in experimental setting, the cases were stained altogether in a single experiment in order to obtain a fully comparable result. Although the number of the cases analyzed in this study is limited, their distribution for sex, age, grade, and stage is representative of usual ccRCC epidemiology. As expected, expression of H3AcK18 and H3AcK14 was limited to the cell nucleus of both clear renal cell carcinoma and epithelial cells of normal kidney (podocytes and epithelial cells of the proximal and distal tubules), whereas KAT3B-p300 antibody stained both the nucleus and the cytoplasm in 42/70 ccRCC cases and 67/69 epithelial cell of the peritumoral normal kidneys. Our results have taken into consideration only the normal kidney or neoplastic epithelial cells whereas endothelial and inflammatory intra and perineoplastic cells, also expressing the three proteins, were carefully excluded. The percentage of tumor or normal epithelial cells expressing the three antibodies was highly variable among the different cases (Additional file 4). Patients’ characteristics according to p300, H3AcK14, and H3AcK18 expression are summarized in Table 2. Overall, there was no significant difference in the frequency of positive cells between the tumor and adjacent normal kidney tissues for all the three antibodies used. The frequency of H3AcK18, H3AcK14, and p300 positive epithelial cells in normal tissues, although different among cases, showed similar mean + SD values independently from the sex, age, grade, and stage of the hosted tumor. Surprisingly, only the G1 tumors showed a significant variation of the three parameters with respect to their normal counterpart, suggesting that G1 tumors may show different pathway of transformation or progression (Fig. 5b). As shown in Fig. 5b and Table 2, G2-G3 ccRCC presented a significant higher expression of p300 and H3AcK18 and a lower expression of H3AcK14 compared to G1 ccRCC (*p* < 0.001). Furthermore, high-stage ccRCCs presented a significant lower expression of H3AcK14 (Table 2). Interestingly, in all the tumor cases, the expression of p300 is directly correlated with H3AcK18 and inversely to H3AcK14, Fig. 5c shows two significant cases. On the multivariable analysis, H3AcK14 and p300 were found to be independent predictors of high-grade ccRCC (Table 3). Particularly, p300 increased the risk of high-grade ccRCC by 7.6% per unit (OR 1.076, IC 1.029–1.236, *p* = 0.001) and H3AcK14 reduced the risk of high-grade ccRCC by 3% per unit (OR 0.971, IC 0.943–0.999, *p* = 0.0041). H3AcK14 was also independent predictors of high-stage ccRCC. Particularly, H3AcK14 expression reduced the risk of stages II–III by 2.5% per unit (OR 0.975, IC 0.953–0.999, *p* = 0.041).

**Table 2:** Distribution of histone H3K18 and K14 acetylation and p300 in ccRCC tumor versus paired normal kidney according to tumor grade and stage [32, 41]

	Normal kidney	ccRCC	*T vsN*	*ccRCC G 1*	*ccRCC G ≥ 2*	*G1* vs *G ≥ 2*	*ccRcc* stage 1	*ccRCC* stage ≥ 2	Stage 1 vs stage ≥ 2
H3AcK14% mean ± SD	38.54 ± 29.33	34.26 ± 30.86	0.319	58.50 ± 24.605	24.56 ± 27.753	0.000	39.59 ± 32.583	24.04 ± 24.775	0.051
Male	38.91 ± 32.31	34.85 ± 30.34	0.52						
Female	37.83 ± 23.36	33.13 ± 32.45	0.65						
M vs F	0.796	0.715							
H3AcK18% mean ± SD	77.02 ± 19.99	68.51 ± 25.57	0.26	48.65 ± 22.335	76.46 ± 22.403	0.000	68.76 ± 25.848	68.04 ± 25.578	0.945
Male	75.83 ± 22.91	66.91 ± 27.03	0.12						
Female	71.58 ± 22.74	71.58 ± 22.74	0.14						
M vs F	0.939	0.546							
p300% mean ± SD	70.46 ± 23.46	67.73 ± 21.17	0.21	46.65 ± 17.388	76.16 ± 16.133	0.000	66.35 ± 22.584	70.38 ± 18.332	0.527
Male	65.67 ± 20.32	72.67 ± 23.82	0.13						
Female	71.67 ± 22.63	66.29 ± 22.66	0.41						
M vs F	0.166	0.189

**Table 3:** Odds ratios (OR) and 95% confidence interval (CI) for predicting G2-G3 ccRCC and stage II–III ccRCC

	Risk of G2-G3 ccRCC			Risk of stage II–III ccRCC		
	OR	95% CI	*p*	OR	95% CI	*p*
Age	0.994	0.926–1.068	0.875	1.054	0.998–1.113	0.062
p300t	1.076	1.029–1.126	0.001	1.003	0.973–1.034	0.851
H3AcK18	1.021	0.987–1.056	0.223	0.981	0.954–1.008	0.170
H3AcK14	0.971	0.943–0.999	0.041	0.975	0.953–0.999	0.041

## Discussion

In the last decades, it has been demonstrated that genes aberrantly regulated in human cancer play a fundamental role in tumor onset and progression. Overall, epigenetic regulation tuning cell differentiation in response to environmental stimuli can be considered a driving alteration of tumor progression and a response sign to therapy. Aberrant patterns of post-translational modifications of histones and cellular proteins lead to alteration of the epigenetic landscape that characterizes human diseases, from cancer to inflammatory and neurological disorders [42, 43]. K-histone acetyltransferases (KATs) are indeed aberrantly expressed in cancer and contribute to oncogenic transformation, thus representing, more importantly, potential targets for therapeutic intervention. KAT3B-p300 is a critical regulator of hematopoiesis, and its heightened expression is recurrent in human malignancies such as prostate [44], liver [12, 45], and breast cancer and is predictive of worse prognosis. Accordingly, the activation of several oncogenes which directly sustain cancer proliferation such as STAT3, NF-κB, and HIF1α are subjected to acetylation. Furthermore, it has been highlighted the role of p300 as a coactivator in the induction of super-enhancers, master hub coordinating the expression of cluster of transcriptional enhancers controlling fundamental gene circuitries responsible for cell identity [46]. p300 is engaged not only in the acetylation of nuclear histones but also of non-histone proteins such as transcription factors involved in autophagy [47], motility, and metastatic processes [48]. Collectively, these reports, as part of a vast literature, suggest many reasons why p300 represents a promising therapeutic target in the treatment of refractory cancer types [49]. Although the mechanisms that regulate p300 activity have not been yet fully highlighted, the importance of p300 intracellular localization for its activity [50–53] has acquired relevance. Lysine acetylation co-regulates several cellular functions through large macromolecular complexes involved in chromatin remodeling, splicing, nuclear transport, and actin nucleation [54]. In pancreatic cancer, a nuclear signaling between Src and p300, with a Src-dependent phosphorylation of p300, regulates gene promoters of AT-hook (HMGA)2, SET, and SMYD3 with effects on cell migration and invasiveness of tumor cells [55]. In the cytoplasm, acetylation increases the stability of actin fibers of the cytoskeleton [56] while opposed deacetylation leads to their destabilization with consequent-reduced migration and motility of cells. In renal clear cell carcinoma 786-O cell line and in papillary thyroid K1, we have demonstrated that KAT inhibitor CPTH2 [23] increases cell death and apoptosis, changes adhesion and cytoskeletal organization, and decreases cell invasiveness and migration. Interestingly, we showed that CPTH2 acts primarily on KAT3B-p300. Although CPTH2 was shown to inhibit GCN5 in human cells, it is also to be underlined that KAT2A-GCN5 expression in renal normal cells and in ccRCC is very low (according to Human Protein Atlas and our unpublished results). Administration of CPTH2 and silencing of p300 showed identical effects in treated cells. We also report that CPTH2 lowers the concentration of p300 at protein level both in the nucleus and in the cytoplasm suggesting that it may carry out several functions in the cell. In the cytoplasm, it may affect actin cytoskeleton stability lowering cell motility while, in the nucleus, it inhibits global levels of histone H3 acetylation and p300 dependent H3AcK18 [43] with several regulatory effects, such as regulation of the expression of genes and oncogenes such as AKT1, TGFb2, and HIF1a. Alteration of post-translational modifications at selected histone N-terminal residues is an emerging novel tool for early diagnosis and prognosis. Oxidative stress induced by malignant transformation of renal tubular epithelium changes global acetylation of histones H3-K9, K18, K27, and K14 [57]. Hypoacetylation of H3AcK18 is associated to prostate carcinoma with poor prognosis [58], and increase of H3AcK18 caused by absence of SIRT7 at sites of DNA damage affects the maintenance of genome integrity [59]. Lower levels of H3K3me2 and H3AcK18 are in fact predictive for higher recurrence in prostate, lung, and kidney cancer patients and can be used for distinguishing clinical outcomes of patients with substantially similar clinico-pathological variables [60]. On the basis of the effects of CPTH2 in lowering p300 and H3AcK18 and the vast literature on the relevance of this histone mark as prognosticator in cancer patients, we wanted to extend the analysis of histone H3 acetylation at K18 and K14 along with p300 in 70 cases of ccRCC tumor patients (listed in Table 1). In the presented study, we started from western blot analysis on global levels of H3AcK18 and H3AcK14 performed by comparison of nuclear extracts from tumor tissue and peritumoral normal epithelium of each single patient, which revealed a great heterogeneity in the global levels of H3AcK14 and H3AcK18 with respect to unmodified H3 among patients. This observation motivated us to extend our study to 70 ccRCC cases grouped for tumor grade. To be sure to exclude from our tissue analysis immunostaining due to non-specific cytoplasm background, we used high dilutions of H3AcK18, H3AcK14 antibodies (methods), and this allowed us to stain, exclusively, cell nuclei and exclude false positive and cross reaction (Fig. 4c, d and Additional file 3). Statistical analysis of our collected results showed that p300 and H3AcK18 levels gave identical profiles supporting selectivity of p300 for H3AcK18 also in ccRCC specimens. Importantly, the analysis showed an identical pattern of p300, H3AcK18, and H3AcK14 in the normal peritumoral tissue of all patients confirming the relevance of analyzing normal epithelium in our screening. Lower p300/H3AcK18 opposed to higher H3AcK14 in low-grade ccRCC, G1 cases with respect to normal epithelium was observed. In addition, with tumor worsening to G2-G3 grade, while p300/H3AcK18 come back to levels found in normal tissues, H3AcK14 showed a significant, progressive decrease (Fig. 5b) as summarized in Table 2. The opposed degree of H3AcK14 vs H3AcK18/p300 expression is significatively restricted to G1 tumors and may therefore represent an important epigenetic signature of low-grade ccRCC. Notably, conflicting results have been reported on the expression levels of histone H3 acetylation and specific H3AcK18 and H3AcK14 in cancer. While conflicting results reported the relevance of the acetylation of H3, H3K18 and H3K14 in ccRCC progression [9, 61] warrant are issued for a more thorough study of ccRCC from different and relevant casuistic and analytical procedures. Collectively, the presented study reports the identification of a novel epigenetic signature for tracing ccRCC tumor tissues based on low-p300/H3AcK18 vs high-H3AcK14 ratio in global histone H3 acetylation, distinctive of low-grade G1 tumors, and prognosticators for tumor aggressiveness. Further molecular analysis for cancer markers such as expression of oncogenes and oncosuppressors or noncoding RNAs can be developed to identify additional characterizing features for the classification of high- or low-H3K18/K14 ratio found in ccRCC low-grade G1 tumors.

## Conclusion

The p300 inhibitor CPTH2 lowers cell invasiveness and viability in ccRCC 786-O cell line. It is a promising compound for counteracting the increase of p300 and H3AcK18 found in higher grade G2, G3 ccRCC tumor tissues. Finally, the opposed ratio of p300-H3AcK18 vs H3AcK14 represents a novel prognosticator signature of low grade, and G1 ccRCC tumors and CPTH2 may efficiently counteract the increasing of both p300 and H3AcK18 in tumor progression.

## Additional files

Additional file 1: Cell cycle progression is not affected by treatment of ccRCC 786-O cell line with CPTH2. FACS analysis of ccRCC 786-O cell line treated with DMSO *w*/*w* CPTH2 (100 μM) at increasing times. Apoptotic profiles of ccRCC 786-O and papillary thyroid K1 cell lines untreated and grown in DMSO *w*/*w* CPTH2 at increasing times. (TIFF 24906 kb)

Additional file 2: 786-O cells grown in DMSO *w*/*w* CPTH2 for 24 and 48 h and immunostained with anti-Ki67 and anti-cyclin D1 show that CPTH2 treatment does not affect cell cycle progression. (TIFF 24905 kb)

Additional file 3: Immunostaining of K1 papyllary thyroid cells with p300 antibody after 72 h of treatment with CPTH2 (100 μM) compared to untreated and DMSO controls. Apoptotic percentage of 786-O cells treated with proteasome inhibitor MG-132 (1 h) and then incubated in DMSO *w*/*w* CPTH2 shows no significative changes of the apoptotic profiles compared to the untreated controls. p300 immunostaining of 786-O cells were pretreated for 1 h with proteasome inhibitor MG132, then grow in DMSO *w*/*w* CPTH2 for 18 h suggest that there is no significative proteolysis of p300 upon inhibition of the proteasome. (TIFF 30444 kb)

Additional file 4: Immunostaining of tissue sections from ccRCC tumor and normal tissues with p300, H3AcK18, and H3AcK14 antibodies. Two opposite cases are shown, patient no. 1 with low p300/H3AcK18 vs. high H3AcK14. Patient no. 41, the opposite, high p300/H3AcK18 vs. low H3AcK14. (TIFF 37242 kb)

## Abbreviations

C646: KAT3B-p300 inhibitor
ccRCC: Clear cell renal carcinoma
CPTH2: Cyclopentylidene-[4-(4-chlorophenyl)thiazol-2-yl)hydrazone
K1: Papillary thyroid cancer cells
KAT: K-Histone acetyltransferase
KAT2A: GCN5
KAT3B: p300
