# Supplementary figures and images for: KAT3B-p300 and H3AcK18/H3AcK14 levels are prognostic markers for kidney ccRCC tumor aggressiveness and target of KAT inhibitor CPTH2
Source: Clin Epigenetics. 2018 Apr 4;10:44. doi: 10.1186/s13148-018-0473-4 (PMC5885315; doi:10.1186/s13148-018-0473-4)

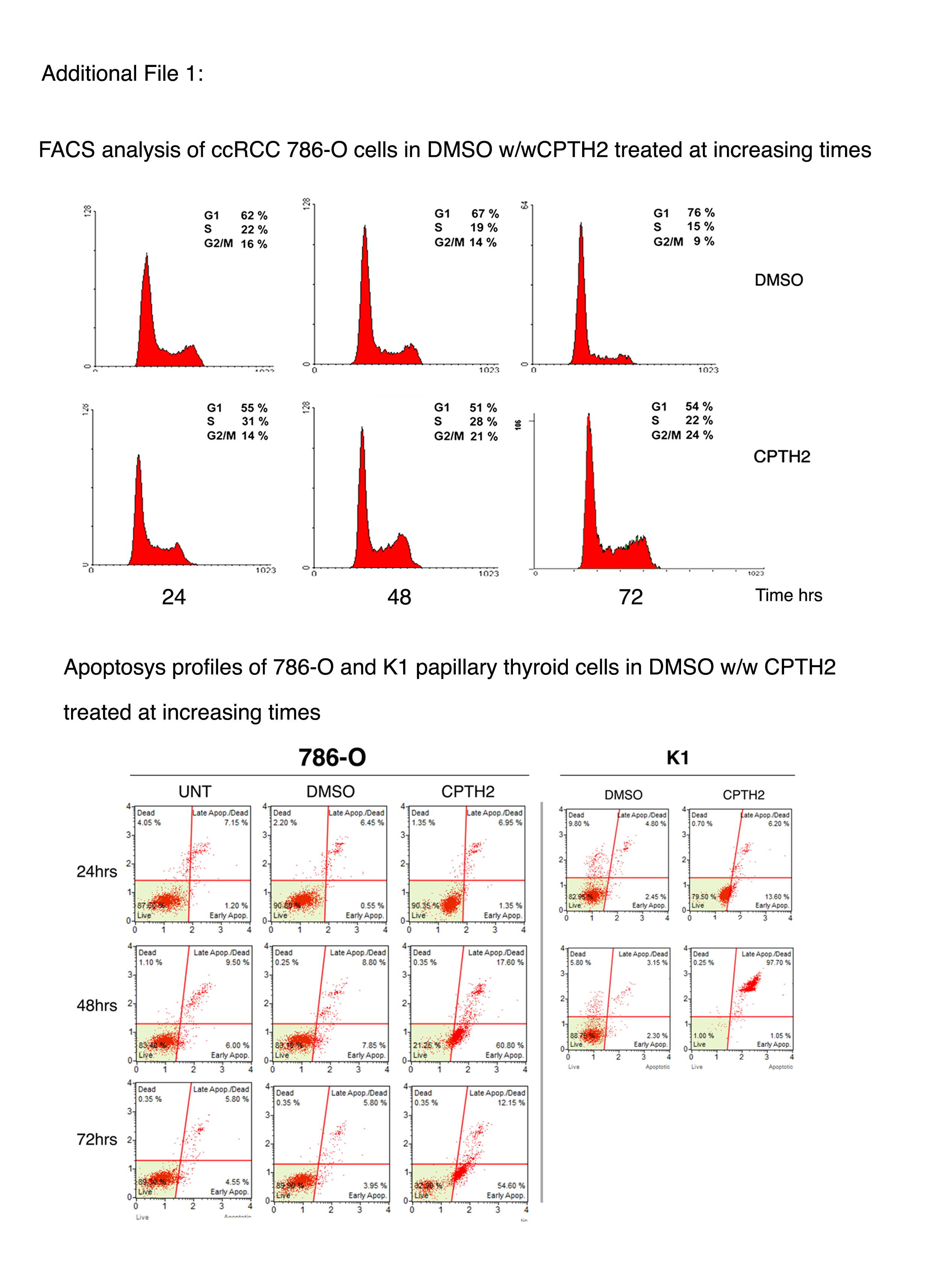

Supplement: Supplementary file 1 — Cell cycle progression is not affected by treatment of ccRCC 786-O cell line with CPTH2. FACS analysis of ccRCC 786-O cell line treated with DMSO w/w CPTH2 (100 μM) at increasing times. Apoptotic profiles of ccRCC 786-O and papillary thyroid K1 cell lines untreated and grown in DMSO w/w CPTH2 at increasing times. (TIFF 24906 kb) [file 13148_2018_473_MOESM1_ESM.tif]

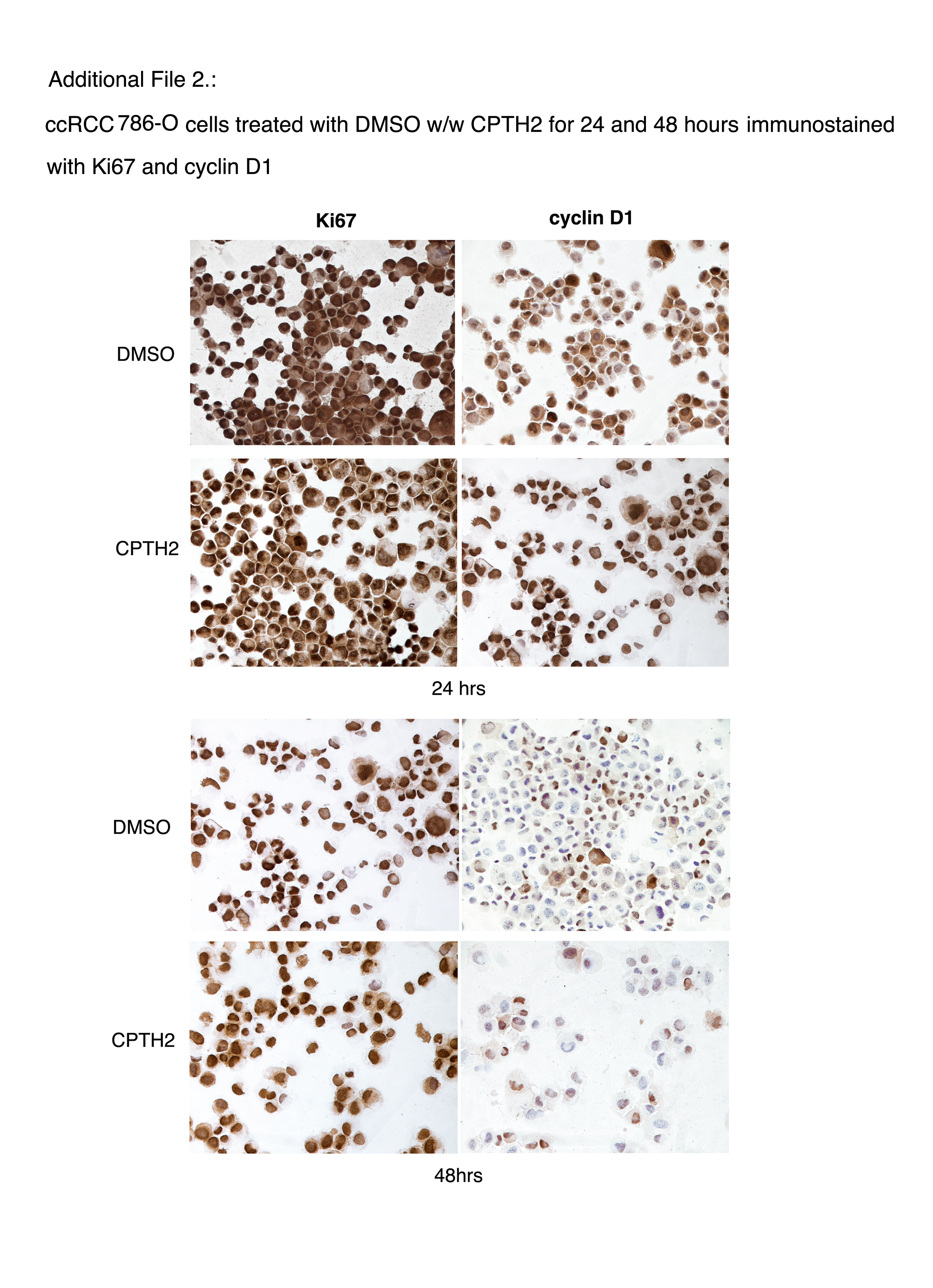

Supplement: Supplementary file 2 — 786-O cells grown in DMSO w/w CPTH2 for 24 and 48 h and immunostained with anti-Ki67 and anti-cyclin D1 show that CPTH2 treatment does not affect cell cycle progression. (TIFF 24905 kb) [file 13148_2018_473_MOESM2_ESM.tif]

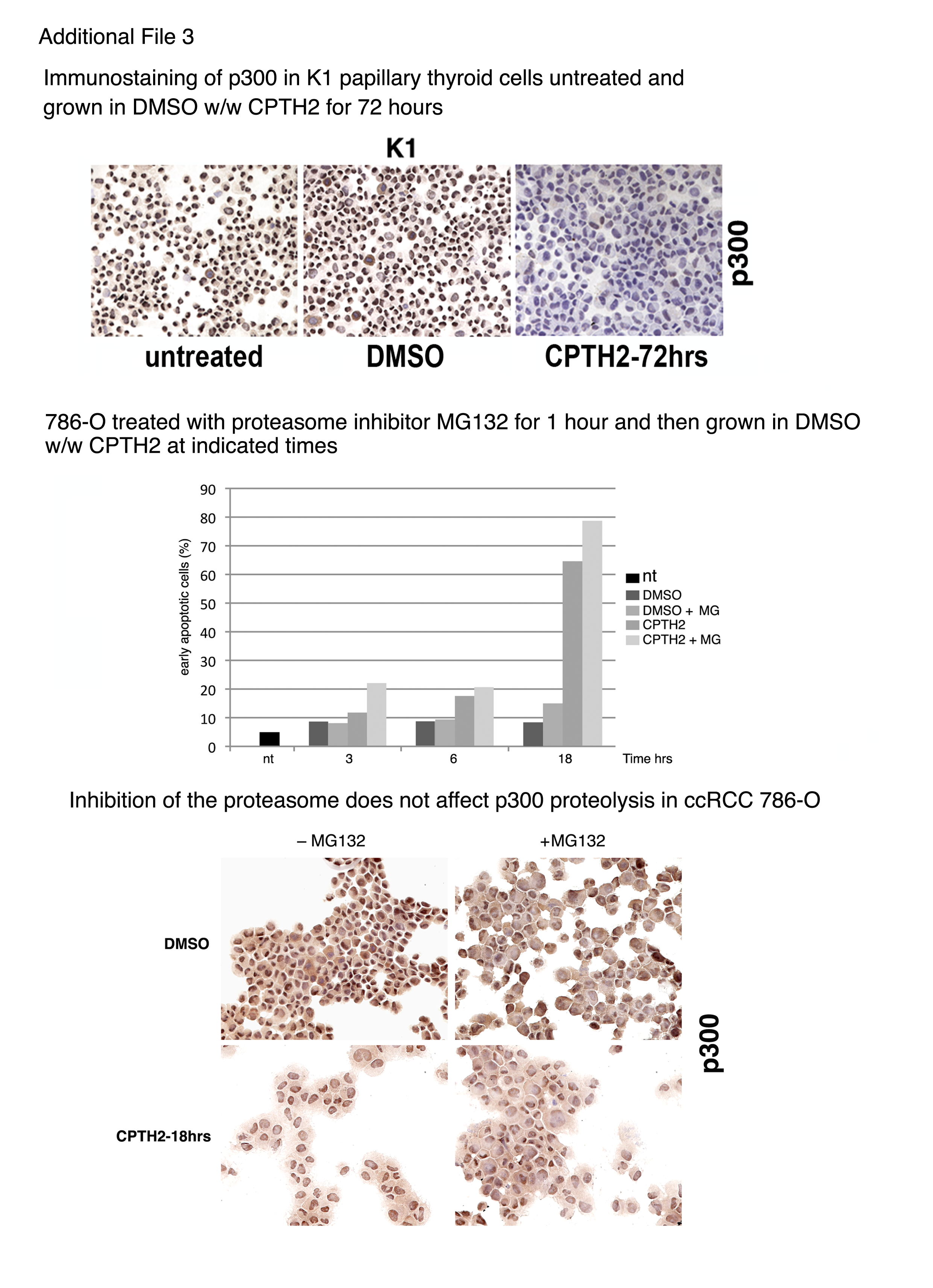

Supplement: Supplementary file 3 — Immunostaining of K1 papyllary thyroid cells with p300 antibody after 72 h of treatment with CPTH2 (100 μM) compared to untreated and DMSO controls. Apoptotic percentage of 786-O cells treated with proteasome inhibitor MG-132 (1 h) and then incubated in DMSO w/w CPTH2 shows no significative changes of the apoptotic profiles compared to the untreated controls. p300 immunostaining of 786-O cells were pretreated for 1 h with proteasome inhibitor MG132, then grow in DMSO w/w CPTH2 for 18 h suggest that there is no significative proteolysis of p300 upon inhibition of the proteasome. (TIFF 30444 kb) [file 13148_2018_473_MOESM3_ESM.tif]

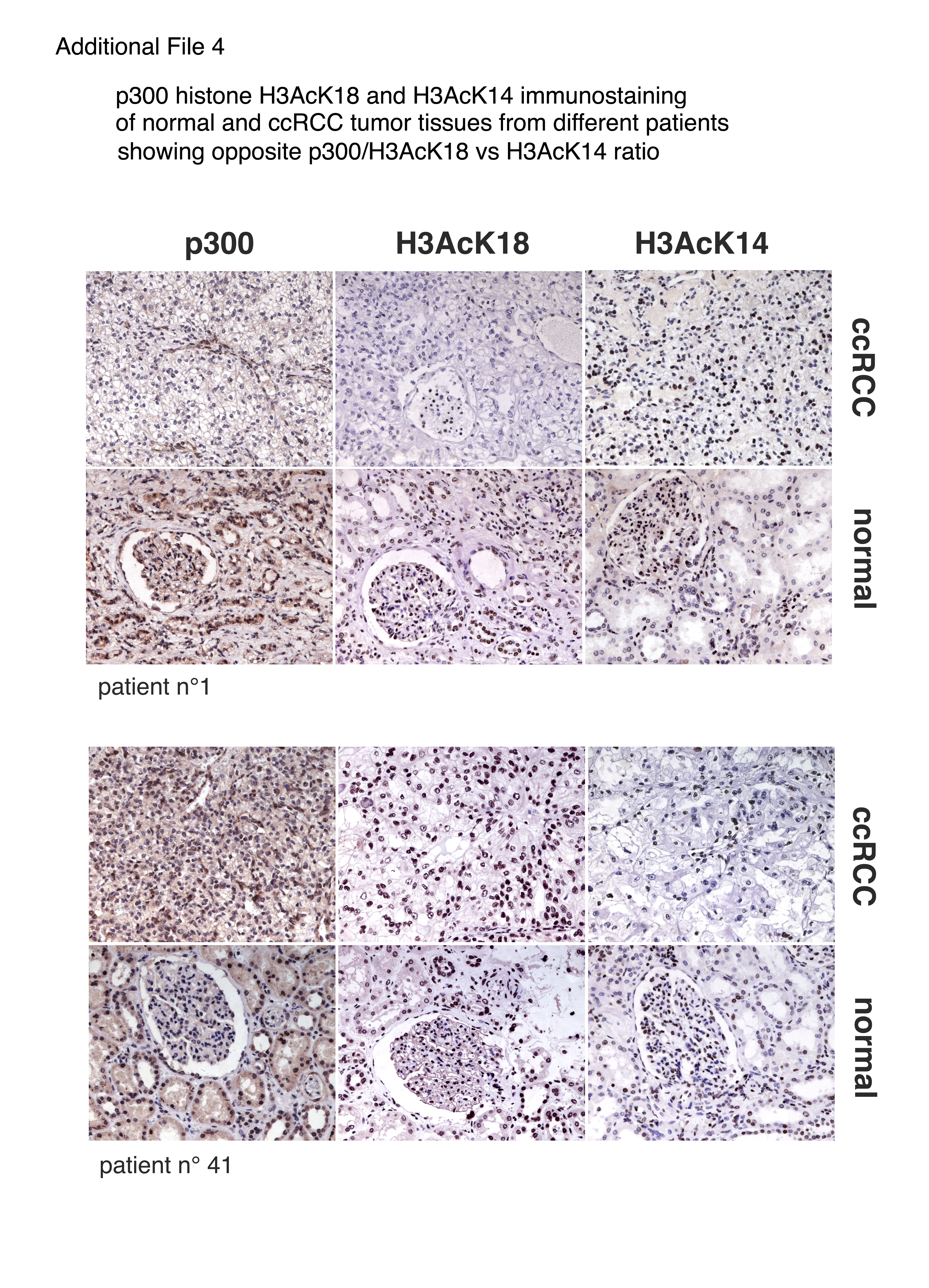

Supplement: Supplementary file 4 — Immunostaining of tissue sections from ccRCC tumor and normal tissues with p300, H3AcK18, and H3AcK14 antibodies. Two opposite cases are shown, patient no. 1 with low p300/H3AcK18 vs. high H3AcK14. Patient no. 41, the opposite, high p300/H3AcK18 vs. low H3AcK14. (TIFF 37242 kb) [file 13148_2018_473_MOESM4_ESM.tif]
